# Hiding in plain sight

**DOI:** 10.1093/hropen/hoad015

**Published:** 2023-05-15

**Authors:** Paul Pirtea, Dominique de Ziegler, James Toner, Richard Scott, Jean-Marc Ayoubi

**Affiliations:** Hospital Foch, Suresnes, France; Hospital Foch, Suresnes, France; Emory University, Atlanta, GA, USA; Reproductive Medicine Associates of New Jersey, Basking Ridge, NJ, USA; Hospital Foch, Suresnes, France

Sir,

We read with interest the article by Melo *et al.* regarding progesterone levels and ART outcomes in all regimens used for priming frozen embryo transfers (FET) (Melo *et al.*, 2022). This article follows a slew of publications on the topic and a meta-analysis conducted by these very authors (Melo *et al.*, 2021). According to the mustered studies, lower serum progesterone levels on, or near, the day of embryo transfer (ET) in women receiving vaginal progesterone are associated with poorer FET outcomes (Melo *et al.*, 2021).

The present article by Melo *et al.* is new in that it studies the links between serum progesterone levels and the outcomes in all forms of treatment used for priming FET (Melo *et al.*, 2022). This also includes the situation when subcutaneous progesterone (Lubion^®^) 25 mg BID is used exclusively (Melo et al., 2022). In this particular case, the study shows a biphasic relationship between serum progesterone levels and FET outcome with a sharp decrease in outcome when serum progesterone exceeds 16.3 ng/ml on the day of ET (Melo *et al.*, 2022). Based on their observation, the authors suggest that serum levels of progesterone that exceed this mark exert a counterproductive effect on embryo implantation. Aside from the weaknesses of their study—for instance, there is no indication as to why patients were prescribed HRT using exclusively subcutaneous progesterone rather than other regimens, and despite limited evidence for biological plausibility and a relatively small cohort (n = 57)—Melo *et al.* conclude that this regimen can harm FET implantation (Melo *et al.*, 2022), which could potentially be misleading for patient and medical care providers.

Melo *et al.* (2022) also stress the fact that their present study is the first on the topic that uses a prospective (though not randomized) design. Yet all studies—retrospective or prospective—rely, for the quality of their conclusion, on the data coming in. Melo *et al.*’s current study, while prospective, is indeed rather complex (Melo *et al.*, 2022). Aside of being prospective and multicentric, it mixes several different protocols for timing FET without indicating the reasons retained by clinicians for choosing a particular approach. Furthermore, it combines single and multiple embryo transfers and, for HRT regimens, limited information on the use or not of prior pituitary desensitization. Specifically, for the topic that attracted our attention, the link between serum progesterone levels and FET outcome in the 57 women primed with subcutaneous progesterone 25 mg BID, the authors do not tell us the motives that led them to opt for this HRT regimen option in these patients.

Melo *et al.*’s findings of FET outcomes when timed with subcutaneous progesterone (Lubion^®^) 25 mg BID (Melo *et al.*, 2022) are extremely puzzling with respect to long established knowledge on progesterone levels and implantation. In the natural cycle, Filicori *et al.* have reported that plasma progesterone reaches 35 ng/ml in the luteal phase of the menstrual cycle: the physiological reference in terms of implantation conditions (Filicori *et al.*, 1984). In HRT cycles used for timing ET emanating from a donor egg, Gibbons *et al.* (1998) reported that serum progesterone levels reach, on average, 89.3 ng/ml in women receiving daily i.m. injections of 50 mg. In this case, pregnancy rates are equivalent to those achieved in women receiving vaginal progesterone (Crinone^®^) 90 mg BID (Gibbons *et al.*, 1998). That the levels of progesterone achieved by i.m. progesterone—several orders of magnitude higher than the 16.3 ng/ml mark implicated as detrimental by Melo (Melo *et al.*, 2022)—are not harmful is corroborated by the fact that the ART centers having the best results in the USA all use i.m. progesterone 50 mg/day for luteal support (Knudtson *et al.*, 2022).

We suspect this is a case of asking too much of a statistical test. To take a sample of 57 cases, adjust for multiple confounders, and apply a quadratic equation that seeks out non-linear trends leads to a risk of the ‘detection’ of a trend that may not reflect biological reality. We therefore believe that the safety of the levels of progesterone achieved by subcutaneous administration of Lubion^®^ 25 mg BID should not have been questioned based on this finding.
